# Low level lead exposure and pregnancy outcomes in an observational birth cohort study: dose–response relationships

**DOI:** 10.1186/s13104-016-2092-5

**Published:** 2016-06-04

**Authors:** Caroline M. Taylor, Kate Tilling, Jean Golding, Alan M. Emond

**Affiliations:** Centre for Child and Adolescent Health, School of Social and Community Medicine, University of Bristol, Oakfield House, Oakfield Grove, Bristol, BS8 2BN UK; School of Social and Community Medicine, University of Bristol, Bristol, UK

**Keywords:** ALSPAC, Lead, Pregnancy, Birth outcomes, Dose–response

## Abstract

**Background:**

National and international guidelines on safe levels for blood Pb in pregnancy focus on a threshold above which exposure is of concern. However, it has recently been suggested that the decrease in birth weight per unit increase in blood Pb is actually greater at lower than at higher concentrations of Pb without evidence of a lower threshold of effect. Our aim was to investigate whether there was evidence for a differential effect of maternal Pb levels on birth outcomes and/or a threshold value for effects.

**Methods:**

Blood samples from pregnant women enrolled in the Avon Longitudinal Study of Parents and Children (ALSPAC) were analysed. Data collected on the infants included anthropometric variables. We fitted adjusted multivariable fractional polynomial models for birth outcomes.

**Results:**

Adjusted models that assumed a linear relationship between untransformed blood Pb and the outcomes provided the best fit: an increase of 1 µg/dl was associated with changes in birth weight of −9.93 (95 % CI −20.27, 0.41) g, head circumference −0.03 (95 % CI −0.06, 0.00) cm and crown–heel length −0.05 (95 % CI −0.10, 0.00) cm.

**Conclusion:**

There was no evidence in this study to suggest a supralinear dose–response relationship or a lower threshold for the effect of maternal blood Pb on birth outcomes. This has implications for consideration of national and international guidelines on levels of concern in pregnancy. Exposure to Pb should be kept as low as possible during pregnancy to minimise adverse outcomes.

## Background

Lead (Pb) is a neurotoxic metal that is widespread in the environment. It readily crosses the placenta [1] and can have adverse effects on birth outcomes, possibly by accumulating in the placenta and causing reduced nutrient transfer and oxidative stress and abnormal function [2, 3]. However, studies on the association of blood Pb (B-Pb) concentrations in pregnancy with birth outcomes have had inconsistent results at all levels of exposure [4–7]. Poor birth outcomes are known to be associated with poor developmental trajectories throughout childhood, as well as with long-term implications for adult health, so the effects of Pb levels need to be characterised to enable delivery of appropriate public health policy and individual healthcare to lead-exposed women and newborn infants.

National and international guidelines on for blood Pb focus on a threshold of concern, although the USA is the only country to publish reference values specifically for pregnant women [8]. It has recently been suggested, however, for Pb concentrations <10 µg/dl the deficit in birth weight per 1 µg/dl increase in B-Pb is greater at lower than at higher concentrations, without evidence of a lower threshold of effect [9]. This is of importance given the high prevalence of low level B-Pb exposure among pregnant women in developed countries [10–13] and the controversy regarding the recommended level of concern for maternal B-Pb [8]. We have previously shown adverse effects of increased maternal B-Pb concentrations on birth outcomes, including birth weight, head circumference and crown–heel length, in a large cohort of pregnant women in the UK (the Avon Longitudinal Study of Parents and Children, ALSPAC) using adjusted linear and logistic regression models [14]. Our aim in the present study was to investigate whether there was evidence for a differential effect and/or a threshold value for effects on birth weight and other birth outcomes in the same cohort using multivariable fractional polynomials (mfp).

## Methods

### The ALSPAC study

The study sample was derived from the ALSPAC study, a population-based study investigating environmental and genetic influences on the health, behaviour and development of children. All pregnant women in the former Avon Health Authority with an expected delivery date between 1 April 1991 and 31 December 1992 were eligible for the study; 14,541 pregnant women were initially enrolled, resulting in a cohort of 14,062 live births [15]. The social and demographic characteristics of this cohort were similar to those found in UK national census surveys [16]. Further details of ALSPAC are available at http://www.bris.ac.uk/alspac. The study website contains details of all the data that are available through a fully searchable data dictionary (http://www.bris.ac.uk/alspac/researchers/data-access/data-dictionary/).

### Ethics approval

Ethics approval for the study was obtained from the ALSPAC Ethics and Law Committee and the Local Research Ethics Committees. Consent for questionnaire completion was implied if the questionnaire was completed and returned to the study office: there was no compulsion to do so, and no reward was given. Analyses of biological samples were only carried out with written permission.

### Collection, storage and analysis of blood samples

Whole blood samples were collected in acid-washed vacutainers (Becton and Dickinson, Oxford, UK) by midwives as early as possible in pregnancy. The median gestational age at the time of blood sampling was 11 weeks (range 1–42 weeks, interquartile range 9–13 weeks). Whole blood samples were stored in the original tube at 4 °C at the collection site before being transferred to the central Bristol laboratory within 1–4 days. Samples were at ambient temperature during transfer (up to 3 h). They were then stored at 4 °C in Bristol until analysis.

Samples were sent by courier to the Centers for Disease Control and Prevention (CDC) where they were analysed for lead using inductively coupled plasma mass spectrometry in standard mode by R. Jones (Bethesda, MD, USA; CDC Method 3009.1) as detailed in Golding, Steer, Hibbeln, Lowery and Jones [17]. The analyses were completed on 4284 women for Pb. One sample had a Pb level below the limit of detection (0.29 µg/dl): this sample was assigned a value of 0.7 times the lower limit of detection (LOD/√2) [18, 19].

### Questionnaires

The mothers received postal self-completion questionnaires during pregnancy. The questionnaires are available from the study website (http://www.bristol.ac.uk/alspac/researchers/resources-available/data-details/questionnaires/). Information on environmental and lifestyle factors included data on self-reported age, parity, highest educational qualification and cigarette smoking.

### Pregnancy outcomes

Newborn head circumference and crown–heel length were measured by trained study staff where the mother gave permission or if these data were missing, the values were extracted from the medical records by trained study staff. Birth weight was derived from obstetric data and from central birth notification data: where values disagreed by <100 g then the lowest value was accepted; if the values disagreed by >100 g then the value was coded as missing. Study staff were blinded to the maternal B-Pb. Length of gestation was based on last menstrual period date, ultrasound assessment or other clinical indicators. Where there was conflict between the maternal report and ultrasound assessment, an experienced obstetrician reviewed the clinical records and made a best estimate.

### Statistical analyses

A total of 4190 singleton live births were included. We fitted multivariable fractional polynomials for continuous birth outcomes (birth weight, head circumference, crown–heel length). Fractional polynomials are a method for fitting more flexible polynomials than the usual simple polynomials, and involve selection from a set of polynomial functions. Multivariable fractional polynomial fitting is based on a closed-test procedure that maintains an overall type 1 error (alpha level) of 0.05 for tests among 44 different combinations [20]. For each outcome, a set of potential confounders in addition to B-Pb are selected to enter the model; those that remain with a significance level of 0.2 are retained [20]. The confounders included: maternal educational attainment, smoking, gestational age (centred at 40 weeks), maternal height and pre-pregnancy weight, and sex of the infant. One or two terms of fractional polynomials were explored in terms of *x*^p^ for B-Pb, where the power p is chosen from −2, −1, −0.5, 0.5, 1, 2, 3 and natural logarithmic transformation [20]. The models were repeated with the exclusion of B-Pb values >10.00 µg/dl to model the effects of relatively low exposure (n = 4175). Sensitivity analysis was conducted by excluding the upper and lower 5 % of B-Pb values. In addition, lowess (locally weighted scatterplot smoother) curves were fitted for the three outcomes: this method fits a smooth curve between two variables [21]. Statistical analysis was done using the mfp and lowess commands in Stata v. 13.

## Results

**Fig. 1:**
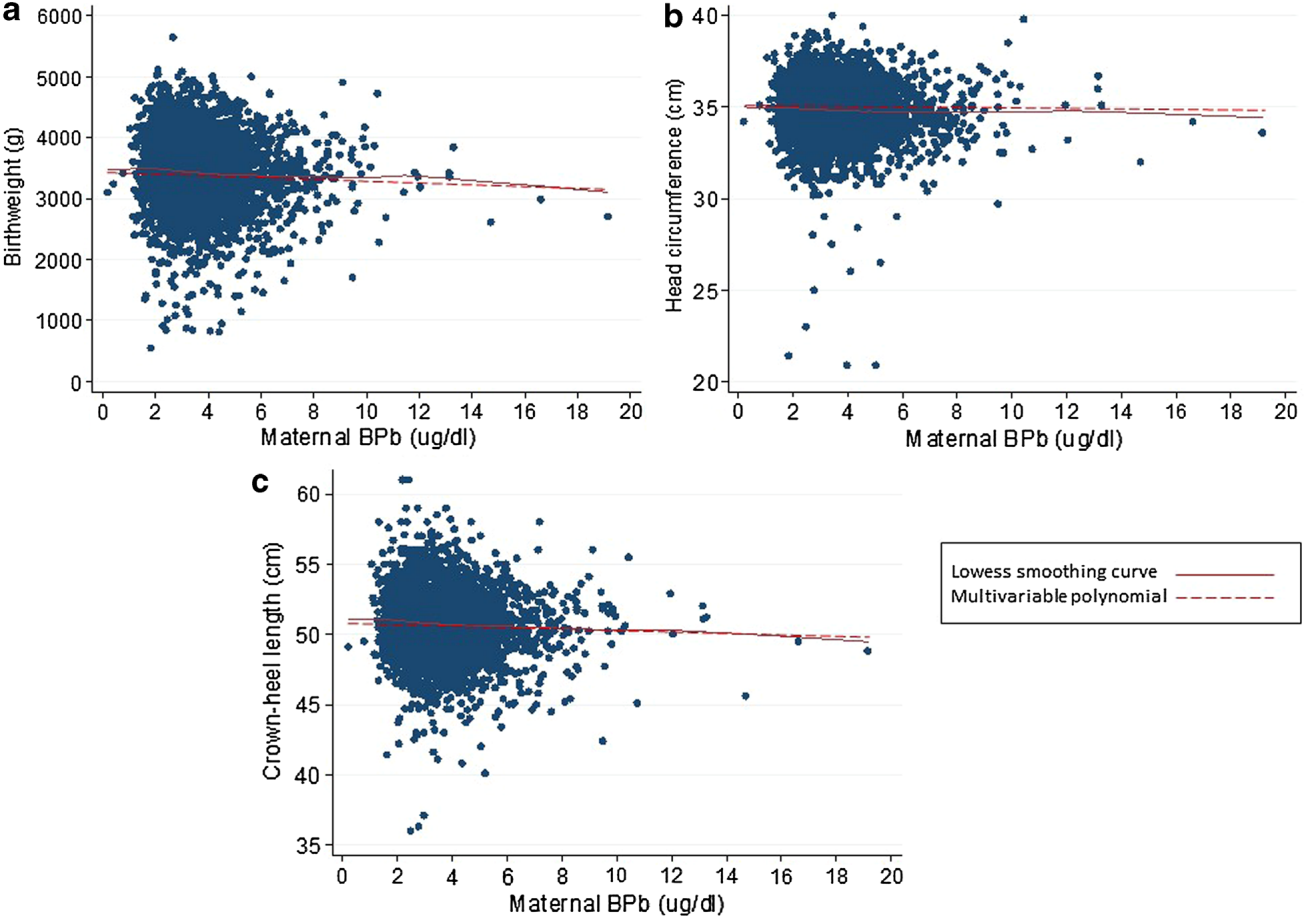
Model-based dose–response relationships between maternal B-Pb levels and birth weight (**a**), head circumference (**b**) and crown–heel length (**c**) fitted using multivariable fractional polynomials (mfp) and lowess smoothing curves. The mfp models were adjusted for maternal educational attainment, smoking, gestational age (centred at 40 weeks), maternal height and pre-pregnancy weight, and sex of the infant. **a** Birth weight, **b** head circumference, **c** crown–heel length

The median B-Pb value was 3.40 (interquartile range 2.66–4.33, range 0.20–19.14, n = 4190) µg/dl. The median value remained as 3.40 (interquartile range 2.66–4.32, range 0.20–9.96, n = 4175) µg/dl when samples >10.00 µg/dl were excluded. The mothers providing a blood sample for Pb analysis were slightly older and had slightly higher educational attainment compared with the rest of the ALSPAC mothers cohort [22]. For all continuous birth outcomes, adjusted models that assumed a linear relationship between untransformed blood Pb and the outcomes provided the best fit; exclusion of values >10.00 µg/dl did not change the fit (all final mfp models: final powers 1) (Table 1; Fig. 1). Sensitivity analysis confirmed that models were robust (linear relationships confirmed; Table 1). Further confirmation was provided by the lowess curves, which were very similar to the mfp fits (Fig. 1).

**Table 1:** Associations between maternal B-Pb and birth outcomes modelled with adjusted multivariable fractional polynomial models in ALSPAC

	n	Unstandardised B regression coefficient (95 % confidence interval)	Fit in adjusted mfp model (final powers)^a^
Adjusted model			
Birthweight (g)	3096	−9.93 (−20.27, 0.41)	1
Head circumference (cm)	2741	−0.03 (−0.06, 0.00)	1
Crown–heel length (cm)	2706	−0.05 (−0.10, 0.00)	1
Adjusted model with exclusion of values >10 µg/dl			
Birthweight (g)	3084	−11.07 (−22.12, 0.18)	1
Head circumference (cm)	2732	−0.04 (−0.07, 0.00)	1
Crown-heel length (cm)	2697	−0.06 (−0.11, 0.00)	1
Adjusted model for sensitivity analysis^b^			
Birthweight (g)	2785	−8.16 (−18.97, 2.65)	1
Head circumference (cm)	2466	−0.04 (−0.08, 0.01)	1
Crown–heel length (cm)	2436	−0.08 (−0.15, 0.00)	1

*mfp* multivariable fractional polynomial

^a^Adjusted for maternal educational attainment, smoking, gestational age (centred at 40 weeks), maternal height and pre-pregnancy weight, and sex of the infant. 1, linear

^b^Exclusion of upper and lower 5 % of B-Pb values

## Discussion

Estimated changes in birth weight, head circumference and crown–heel length with a 1 µg/dl change in blood Pb did not vary across the Pb distribution for the whole sample (values <20 µg/dl). Similar results were obtained for the subsample including values <10 µg/dl. Thus there was no evidence in this study to suggest a supralinear dose–response relationship. There was no evidence to support a lower threshold for the effect of maternal B-Pb on these birth outcomes.

Although supralinear relationships have been noted in the relationship between Pb levels in children and intellectual development [23, 24], most studies that have modelled maternal B-Pb concentrations with birth outcomes have used multiple linear and/or logistic regression to model linear relationships. To our knowledge, there is only a single study that has used multiple linear regression with fractional polynomials, which enables curves to be fitted, to assess continuous birth outcomes [9]. Zhu et al. studied a population taken from the New York Heavy Metals Registry, which includes test reports on Pb concentrations from pregnant women aged 15–49 years living in New York State. Their sample included more than 44,000 live singleton births with a mean maternal Pb concentration of 2.1 µg/dl (median 2.0 µg/dl) reported to the registry in 2003–2005 (participants with values >10 µg/dl were excluded from the analyses). In contrast to our results, it was found that a model assuming a linear relationship between the square root of the B-Pb fitted the data for birth weight better than any other combination of fractional polynomials evaluated: the estimated changes in birth weight with a 1 µg/dl change in B-Pb varied across the B-Pb distribution consistent with the supralinear shape of the dose–response curve. Thus, at the lower end of the distribution, a 1 unit change in B-Pb from 0 to 1 µg/dl was associated with a decrease on birth weight of 27 g, whereas a change from 9 to 10 µg.dl was associated with a decrease of only 4.4 g. In the present study, the decrease was 9.93 g per 1 unit change at all concentrations of B-Pb, ranging from 0.20 to 19.14 µg/dl. Repetition of the analyses with the exclusion of values >10 µg/dl to reflect effects of low exposure explored in the New York State cohort did not change the fit of our models. There are several possible explanation for this difference in findings. First, the New York State cohort study was able to include a far greater number of participants than in our study (45,000 vs 4190 respectively). Second, there are several differences in the population samples. Whereas our study broadly reflected that of the UK population [16], the New York sample included participants who were more likely to be screened because of being at risk for adverse pregnancy outcome or lead exposure. In addition, the New York sample included a high proportion of African American women, whereas our sample was predominantly white (about 97 % of mothers providing a blood sample for analysis were white [22]), and the B-Pb values were slightly lower (median 2.0 µg/dl compared with median 3.4 µg/dl in the present study). Despite these differences, like Zhu et al. we did not find any evidence of a lower threshold of effect.

There are several limitations to this study, which have been described in detail previously [14]. In brief, these include possible sample bias and the inability to take all confounders into account. In addition, the deficits in the birth outcomes are relatively small, and for birth weight, the confidence interval were wide and included zero, suggesting that there could be no overall association using this type of statistical analysis and that the power was relatively low.

## Conclusion

There was no evidence to suggest a supralinear dose–response relationship between maternal B-Pb and birth outcomes. As there was no evidence to suggest a lower threshold for the effect of maternal B-Pb on birth outcomes in this study, exposure to Pb should be kept as low as possible during pregnancy to minimise adverse outcomes. These results are of importance in the consideration of the levels of concern in pregnancy. Investigations in other cohorts are needed.

## Abbreviations

ALSPAC: Avon Longitudinal Study of Parents and Children
B-Pb: blood lead level
